# Shock formation and structure in magnetic reconnection with a streaming flow

**DOI:** 10.1038/s41598-017-08836-8

**Published:** 2017-08-18

**Authors:** Liangneng Wu, Zhiwei Ma, Haowei Zhang

**Affiliations:** 1 College of Sciences, China Jiliang University, Hangzhou, 310018 China; 2 Institute for Fusion Theory and Simulation, Department of Physics, Zhejiang University, Hangzhou, 310027 China

## Abstract

The features of magnetic reconnection with a streaming flow have been investigated on the basis of compressible resistive magnetohydrodynamic (MHD) model. The super-Alfvenic streaming flow largely enhances magnetic reconnection. The maximum reconnection rate is almost four times larger with super-Alfvenic streaming flow than sub-Alfvénic streaming flow. In the nonlinear stage, it is found that there is a pair of shocks observed in the inflow region, which are manifested to be slow shocks for sub-Alfvénic streaming flow, and fast shocks for super-Alfvénic streaming flow. The quasi-period oscillation of reconnection rates in the decaying phase for super-Alfvénic streaming flow is resulted from the different drifting velocities of the shock and the X point.

## Introduction

Magnetic reconnection as a fundamental process in space and laboratory plasmas is widely used to explain the transfer from magnetic energy to kinetic and thermal energies. Many eruptive physical phenomena in magnetized plasmas such as solar flare, magnetospheric substorm, and major disruption in tokamak experiments^1–5^ are considered to be closely related with magnetic reconnection.

Based on resistive magnetohydrodynamic (MHD) model, MHD shocks associated with magnetic reconnection have been widely studied theoretically and observationally in the past decades^6–11^. Three types of MHD shocks (fast shock, intermediate shock, and slow shock), existing in the framework of compressible MHD, have been surveyed in geomagnetic and interplanetary spaces. Lin *et al*.^8^ found that in magnetic reconnection, steady intermediate shocks, slow shocks, slow expansion waves, or contact discontinuity could be generated without a guide field. With a guide field, time-dependent intermediate shocks replace steady intermediate shocks inside the reconnection layer. Hsieh *et al*.^11^ found that the generation of plasma jets and plasma bulges in magnetic reconnection could result in fast shock formation on the flanks of the bulges.

The fast shear flow exists in solar wind, magnetopause boundary, etc. The shear flow can exert conspicuous effects on both magnetic reconnection and shock generation^12–21^. The role of a sub-Alfvénic shear flow in magnetic reconnection is very different from that of a super-Alfvénic shear flow. With the sub-Alfvénic flow, the tearing mode instability plays a dominant role while the Kelvin-Helmholtz instability becomes crucial with the super-Alfvénic flow. For the sub-Alfvénic shear flow, *Belle-Hamer et al*.^16^ found that intermediate shocks and weak slow shocks could emerge along the separatrices. Li *et al*.^21^ also observed the formation of slow shocks in the inflow region. With inclusion of Hall effects, it is found that magnetic reconnection can be stabilized or destabilized by a shear flow under different plasma betas and shear flow widths^20^. A super-Alfvénic shear flow could lead to formation of fast shock in the inflow region^18^.

For a hyperbolic tangent shear flow parallel to magnetic field, the effects on magnetic reconnection have been widely reported as above mentioned. However, bulk plasma flows observed in the magnetotail usually appear as streaming flows that are confined inside the neutral sheet^10, 22, 23^. The influences of these streaming flows on magnetic reconnection have not been studied well. It is suggested in the previous works^24, 25^ that both sub-Alfvénic and super-Alfvénic streaming plasma flows may increase the growth rate of the tearing mode. When the streaming flow thickness is comparable to the current sheet thickness, the growth rate is scaled as from S^−3/5^ to S^−1/2^ (where S is the Lundquist number) for the constant Ψ case, and as S^−1/3^ for the non-constant Ψ case. Sato and Walker^26^ found that the tearing mode is excited much more violently with streaming flow than without streaming flow in the plasma sheet. These previous works indicate that the tearing mode instability in the linear growth stage can be suppressed or accelerated by the streaming flow with different shear thicknesses and velocities.

In this paper, we extend the study of refs 24–26 to investigate roles of sub (super)-Alfvénic streaming flows on magnetic reconnection in the nonlinear growth phase. We mainly focus on the dynamic evolution of shocks and magnetic reconnection with sub (super)-Alfvénic streaming flows based on compressible resistive MHD. It is found that the slow (fast) shocks are observed for sub-(super-) Alfvénic streaming flow in the inflow region or outside of the reconnection separatrices. The time evolution of reconnection rate exhibits a quasi-periodic oscillating behavior for super-Alf vénic streaming flow.

## Methods

With a streaming flow inside the current sheet, we adopt the two-dimensional (2D) compressible resistive MHD model in the Cartesian coordinate system to investigate the generation of shocks in magnetic reconnection. With the 2D model, we have the magnetic field ${\bf{B}}=\hat{y}\times \nabla \psi$, where $\psi (x,z)$ is the magnetic flux function. The following compressible resistive MHD equations are used in the simulation^21^,

1 $$\frac{\partial \rho }{\partial t}=-\nabla \cdot (\rho {\bf{v}})$$

2 $$\frac{\partial (\rho {\bf{v}})}{\partial t}=-\nabla \cdot [\rho {\bf{v}}\,{\bf{v}}+(p+{B}^{2}/2){\bf{I}}-{\bf{BB}}]+{\nabla }^{2}({\bf{v}}-{{\rm{v}}}_{i})/{S}_{v}$$

3 $$\frac{\partial \psi }{\partial t}=-{\bf{v}}\cdot \nabla \psi +({J}_{y}-{J}_{y0})/S$$

4 $$\frac{\partial {B}_{y}}{\partial t}=-\nabla \cdot ({B}_{y}{\rm{v}})+{\bf{B}}\cdot \nabla {v}_{y}+{\nabla }^{2}{B}_{y}/S$$

5 $$\frac{\partial p}{\partial t}=-\nabla \cdot (p{\bf{v}})-(\gamma -1)p\nabla \cdot {\bf{v}}+({J}^{2}-{J}_{0}^{2})/S$$

where **v**, **J**, ρ, p, and **I** are the plasma velocity, the current density, the plasma density, the thermal pressure, and the unit tensor, respectively. The specific heat ratio γ is chosen to be 5/3. **v**_i_ and ${J}_{0}={J}_{y0}$ are the initial velocity and current density, respectively. All variables are normalized as follows: ${\rm{B}}/{{\rm{B}}}_{0}\to {\rm{B}}$, ${\rm{x}}/a\to {\rm{x}}$, ${\rm{t}}/{\tau }_{A}\to {\rm{t}}$, ${\rm{v}}/{{\rm{v}}}_{A}\to {\rm{v}}$, $\psi /({B}_{0}a)\to \psi$, $\rho /{\rho }_{0}\to \rho$, $p/({B}_{0}^{2}/4\pi )\to p$, where ${\tau }_{A}=a/{v}_{A}$ is the Alfvénic time, ${v}_{A}={B}_{0}/{(4\pi {\rho }_{0})}^{1/2}$ is the Alfvénic speed, $a=5{d}_{B}$ and ${d}_{B}$ is the half width of the initial current sheet. $S={\tau }_{R}/{\tau }_{A}$ is the Lundquist number and ${S}_{v}={\tau }_{v}/{\tau }_{A}$ is the Reynolds number, where ${\tau }_{R}=4\pi {a}^{2}/\eta {c}^{2}$, ${\tau }_{v}=\rho {a}^{2}/v$, *c* is the speed of light, *η* is the resistivity, and *v* is the viscosity.

The set of equations (1–5) are solved by Runge-Kutta scheme with fourth-order accuracy in time and in space. The simulation box is ${L}_{x}={L}_{z}=[-2,2]$, with 501 × 1001 grid points that are uniform in both the *x* direction and the z direction. Periodic and free boundary conditions are employed at $x=\pm {L}_{x}$ and $z=\pm {L}_{z}$, respectively. From the condition of initial force-balanced equilibrium, we have the thermal pressure,

6 $$p=(1+\beta ){B}_{0}^{2}/2-{B}^{2}/2$$

where *β* is the asymptotic plasma beta. The initial magnetic field and streaming flow are chosen to be:

7 $${{\bf{B}}}_{i}=[{B}_{0}\,\tanh (z/{d}_{B}),0,0]$$

8 $${{\rm{v}}}_{i}=[{v}_{0}\,sec\,{h}^{2}(z/{d}_{v}),0,0]$$

where *d*_*B*_ and *d*_*v*_ are the half width of the current sheet and the streaming flow, respectively. *B*_0_ and *v*_0_ represent the initial strengths of the magnetic field and the streaming flow, respectively. The parameters are chosen as ${d}_{v}={d}_{B}=0.2$, ${B}_{0}=1.0$, the plasma density ρ = 1, and the plasma beta *β* = 1. In all simulations, the resistivity and the viscosity are assumed to be uniform, i.e., S = S_v_ = 10000.

A small perturbation of the magnetic field is imposed to trigger an onset of the tearing mode instability,

9 $$\delta \psi =\delta {\psi }_{0}\,{\rm{c}}{\rm{o}}{\rm{s}}(\pi x/{L}_{x}){\rm{c}}{\rm{o}}{\rm{s}}(\pi z/2{L}_{z})$$

where $\delta {\psi }_{0}=0.001$. The maximum velocities of the initial streaming flows are set to be ${v}_{0}=0.8$ and 2.0 in our simulations.

The magnetic reconnection rate *R* is defined by:

10 $$R=\eta [J(px)-J(po)]$$

where J is the current density, *px* and *po* are the positions of the X and O points, respectively. The perturbed vorticity in the *y* direction is defined as

11 $${\rm{\Omega }}={\{\nabla \times ({\bf{v}}-{{\bf{v}}}_{i})\}}_{y}$$

where **V**_i_ is the initial velocity of streaming flow in equation (8).

## Results and Discussion

### Sub-Alfvenic Streaming Flow

**Figure 1:**
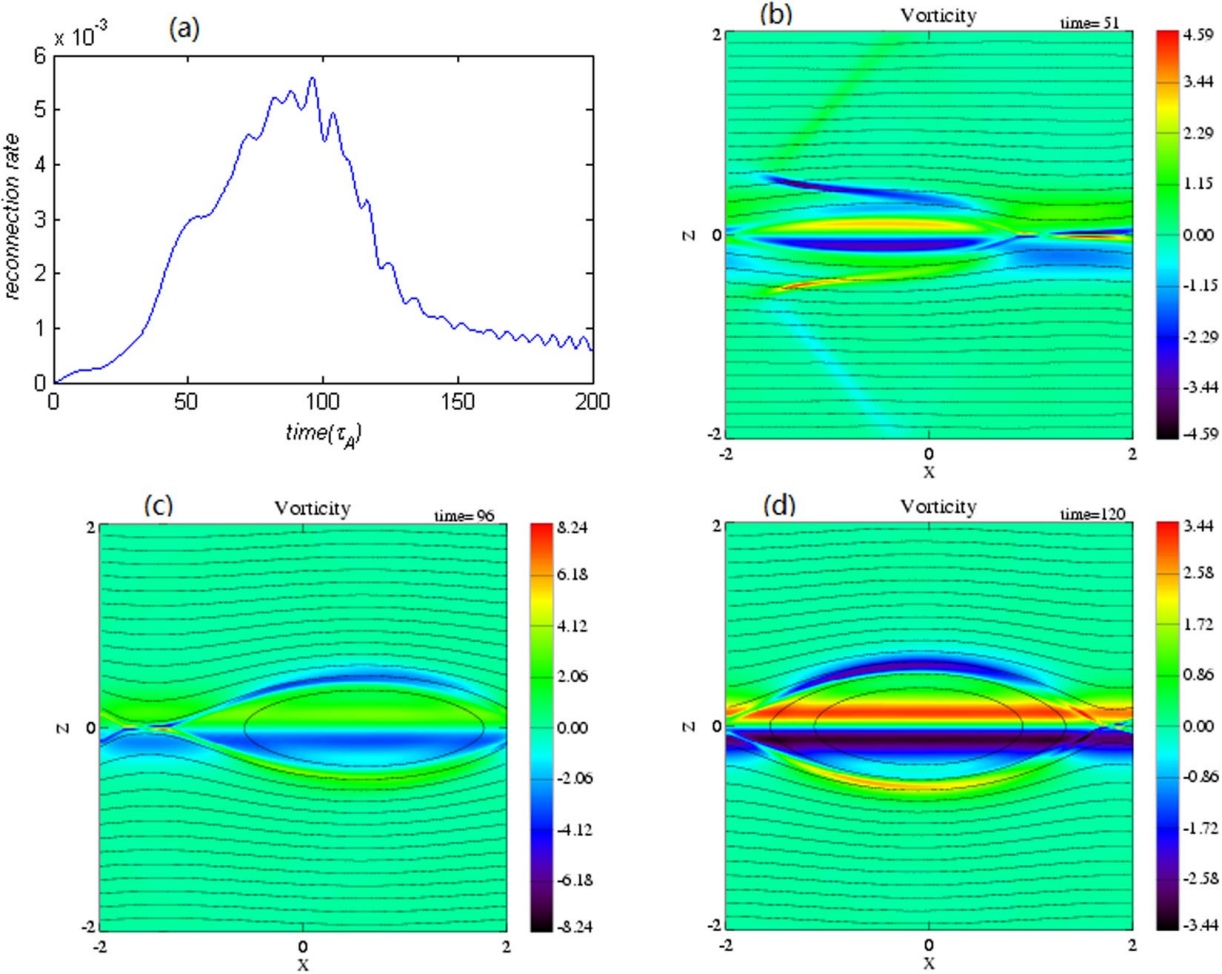
(**a**) Time evolution of reconnection rate for v_0_ = 0.8 and the color contours (**b**,**c** and **d**) for distributions of the perturbed vorticity with magnetic field lines (black solid lines) at three different stages t = 51, 96, 120.

The time development of reconnection rate and the snapshots of perturbed vorticities are shown in Fig. 1. During magnetic reconnection, there are three different phases: the “nonlinear growth phase”, the “phase with maximum reconnection”, and the “decaying phase”. In the nonlinear growth phase, perturbations are mainly confined inside the outflow regions and a pair of discontinuity layers emerges along the separatrices as shown in Fig. 1b. At the same time, weak discontinuity layer exists in the inflow region. With further development of magnetic reconnection, the discontinuity layers disappear gradually in the inflow region, as given in Fig. 1c and d, which is quite different from that with a sub-Alfvenic shear flow^21^. With sub-Alfvénic shear flow, Li *et al*.^21^ found that two pairs of slow shocks are formed in the inflow region away from the reconnection separatrices and last for a long time.

**Figure 2:**
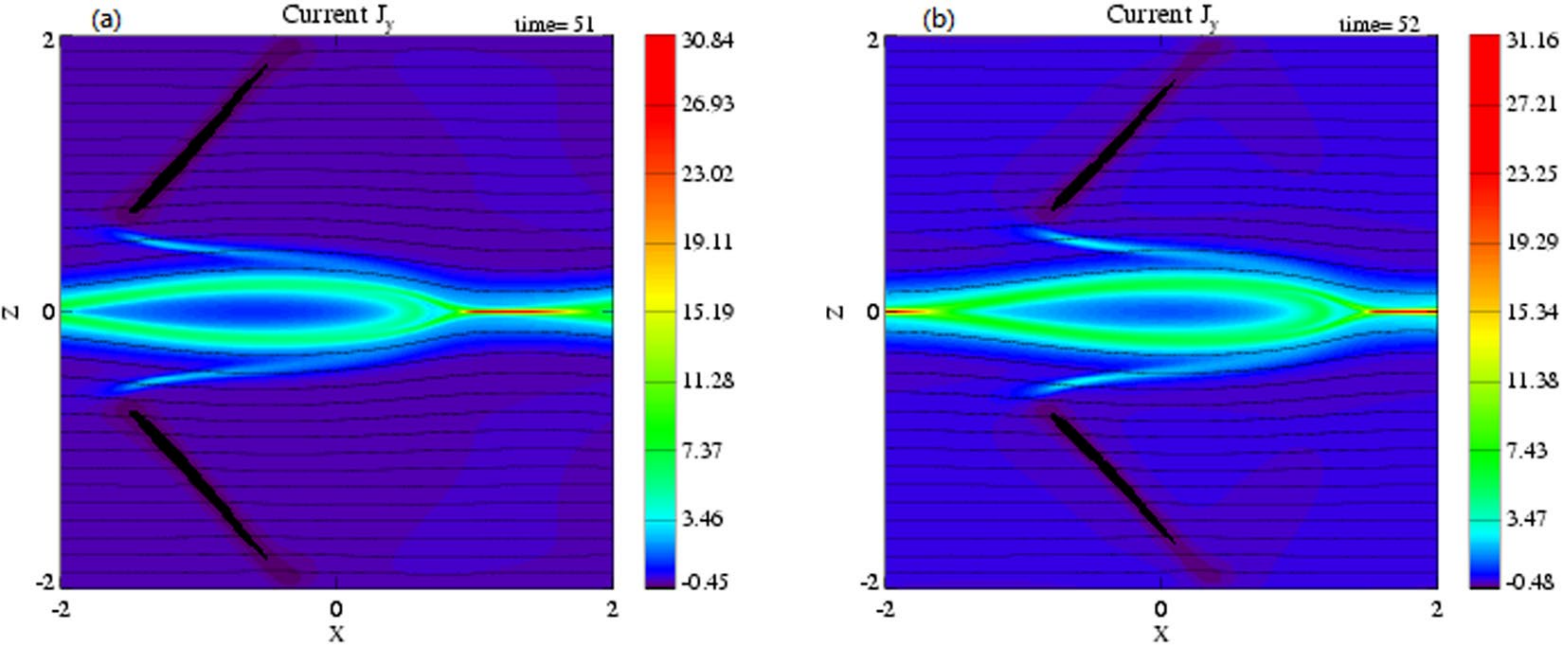
Color contours of the current density (J_y_) with magnetic field lines (black solid lines) at (**a**) t = 51 and (**b**) t = 52.

In order to clarify the evolution features of the discontinuities at the nonlinear growth phase, distributions of the current density (J_y_) at t = 51 and 52 are given in Fig. 2. It is evidently seen that the discontinuities and the magnetic island drifts in the *x* direction. The drift speed of the discontinuity in the inflow region is obtained by examining the time evolution of the x position for maximum dB_z_/dx at a fixed z. At t = 51, the drift speed (v_d_) of the discontinuity in the inflow region is about v_d_ = 0.7v_A_. We assume that the motion of the discontinuity layer in the z direction can be neglected.

**Figure 3:**
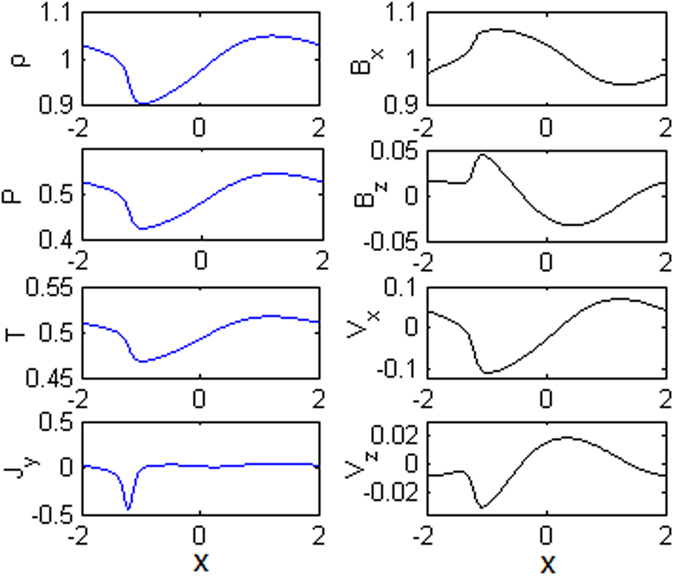
The profiles of the plasma density (ρ), the plasma pressure (P), the plasma temperature (T), the current density (J_y_), the magnetic field (B_x_ and B_z_), and plasma velocity (v_x_ and v_z_) along the x axis at z = 1 at t = 51.

Figure 3 shows the profiles of different physical quantities along the ***x*** axis at z = 1 for t = 51. It is obviously seen that most quantities exhibit large changes about x = −1.2. In order to identify the properties of the discontinuity, the Rankine-Hugoniot relations are used to examine the jumping conditions. Since the Rankine-Hugoniot relations are obtained with the ideal steady state assumption, we need to transform the variables in the upstream and downstream regions into the De Hoffmann-Teller frame^27^ from our simulation frame. Table 1 presents the measured values of variables in the upstream and downstream near the discontinuity, and that calculated from the Rankine-Hugoniot relations in the downstream.

**Table 1:** The variables from the Rankine-Hugoniot Relations for the shock at z = 1 for t = 51 in Fig. 1(b).

	*ρ*	*P*	*B*_*n*_	*B*_*t*_	*v*_*n*_	*v*_*t*_
Upstream	0.9064	0.4246	0.8451	0.6447	0.6442	−0.4868
Downstream	0.9515	0.4603	0.8325	0.6199	0.6077	−0.4481
R-H matching	0.9665	0.4731	0.8451	0.6049	0.6040	−0.4283
Error	1.58%	2.19%	1.51%	2.42%	0.61%	4.42%

The square bracket [F] = F_2_ − F_1_ is used to present the jump of a physical quantity between the upstream (subscript 1) and downstream (subscript 2). The error is defined as $|{F}_{2}-{F}_{R}|/|{F}_{2}|$, where F_R_ is the value calculated from the Rankine-Hugoniot relations in the downstream. The normal components of the magnetic field (*B*_*n*_) and the velocity (*V*_*n*_) are perpendicular to the shock plane. The tangential components of magnetic field (*B*_*t*_) and velocity (*V*_*t*_) are parallel to the shock plane. The angle of the discontinuity relative to the x axis at time t = 51 is about 55^0^ from Fig. 2. The characteristic speeds of the slow, intermediate, fast mode are, respectively, v_sl1_ = 0.6096, v_I1_ = 0.8877, and v_F1_ = 1.2867 in the upstream, and v_sl2_ = 0.6119, v_I2_ = 0.8534, and v_F2_ = 1.2501 in the downstream. Comparing with the normal components of the plasma flow velocity with respect to the discontinuity in the upstream and downstream in Table 1, we find v_sl1_ < v_n1_ < v_I1_, v_n2_ < v_sl2_. The other variables satisfy $[\rho ] > 0,[P] > 0,[{v}_{n}] < 0,[|{B}_{t}|] < 0$. Therefore, we can deduce that the discontinuities in the inflow region in Fig. 1b are the slow shocks.

### Super-Alfvenic Streaming Flow

In ideal MHD, plasma motion across magnetic field lines is not allowed. Any bending of magnetic field lines inside the current sheet changes the size of the streaming flow channel. Consequently, the streaming flow velocity decreases/increases with increase/decrease of the flow channel size along the current sheet if plasma compressibility is ignorable. In the region with inward bending field lines, the plasma pressure based on Bernoulli’s Equation decreases due to increase of the streaming flow velocity, which causes further bending of magnetic field lines. We conclude that the sausage instability due to a streaming flow in the current sheet accelerates thinning of the current sheet or boosts the tearing mode instability^24^.

**Figure 4:**
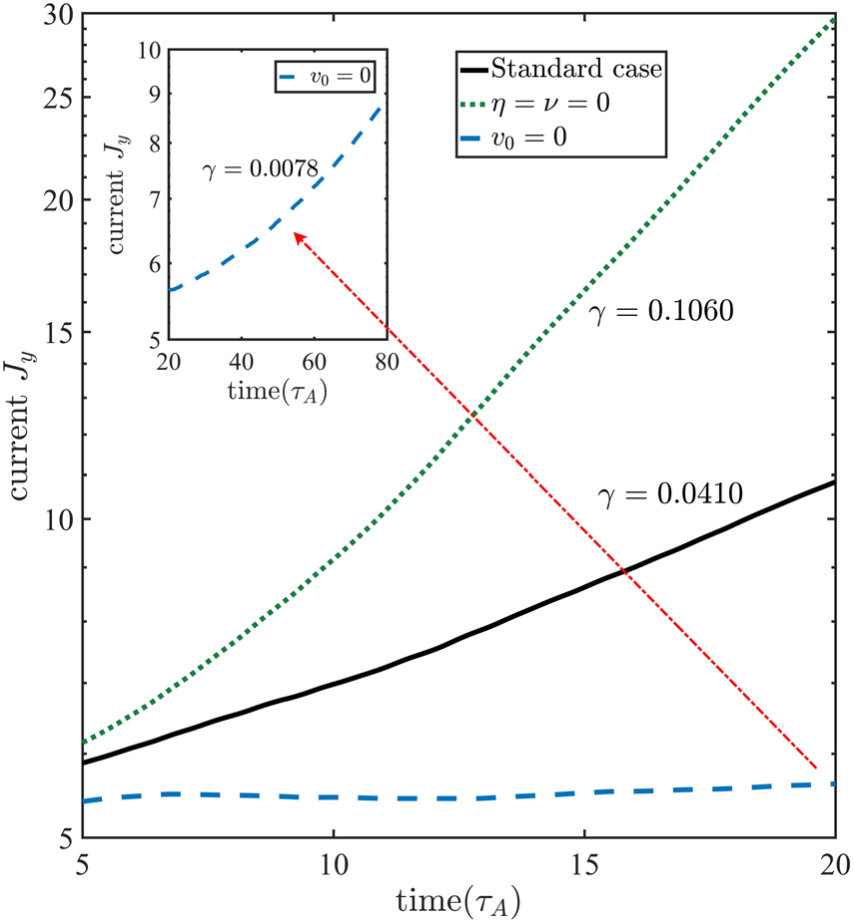
Time evolutions of the current density in the linear phase for three different cases: magnetic reconnection with streaming flow (solid line) and without streaming flow (dashed line), no reconnection with streaming flow (dotted line).

Figure 4 shows the time evolutions of the current density and the linear growth rates for three different cases: magnetic reconnection with streaming flow v_0_ = 2.0 (solid line) and without streaming flow (dashed line), no reconnection with streaming flow (dotted line). It is evident that the growth rate of the sausage instability is much larger than that of the tearing mode instability. Thus, the sausage instability will drive quickly thinning of current sheet that accelerates development of magnetic reconnection. Indeed, the growth rate of the tearing mode instability with super-Alfvénic streaming flow is almost five times larger than that without streaming flow.

**Figure 5:**
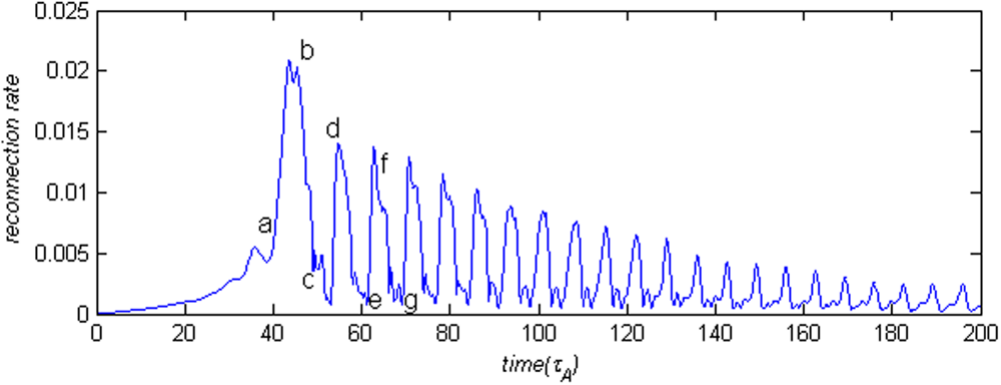
Time evolution of reconnection rate for v_0_ = 2.0 case.

Figure 5 shows the time evolution of reconnection rate with the super-Alfvénic streaming flow v_0_ = 2.0. It can be seen that magnetic reconnection develops much faster with the super-Alfvénic streaming flow than with the sub-Alfvénic streaming flow in Fig. 1a because the growth rate of the sausage instability increases with increase of the streaming flow velocity. The time to reach the maximum reconnection rate only takes about 40 ${\tau }_{A}$ for the super-Alfvénic streaming flow while it takes about 100 ${\tau }_{A}$ the sub-Alfvénic streaming flow. The maximum reconnection rate is also nearly four times larger than that in Fig. 1. The dynamics of magnetic reconnection with super-Alfvénic streaming flow exhibits qualitative difference from that with sub-Alfvénic flow for the decaying phase. The reconnection rate shows a quasi-period oscillating decay with time.

**Figure 6:**
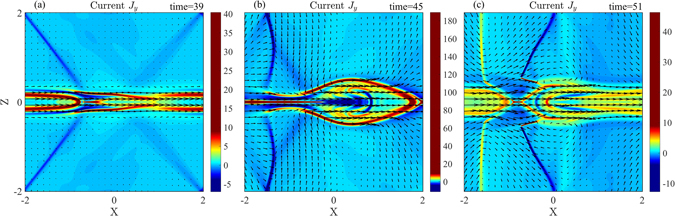
Color contours of the current density (J_y_) with magnetic field lines (black solid lines) and flow vectors (black arrow) for the streaming flow v_0_ = 2.0 at (**a**) t = 39, (**b**) t = 45, and (**c**) t = 51, corresponding to a, b, c labeled in Fig. 5, respectively.

The current density (J_y_) distributions at three different times are shown in Fig. 6. In Fig. 6(a), there are a pair of strong discontinuity and two pairs of weak discontinuity in the inflow region. While the strong discontinuity is propagating in the positive *x* direction, its orientation changes due to one side of the discontinuity connecting to a slow drifting discontinuity that is located near the separatrix. The discontinuity gradually becomes perpendicular to the *x* axis as in Fig. 6(b). Later, a new pair of the discontinuity with a positive current density emerges. Thus, there are two pairs of discontinuities appeared around the X point in the inflow region in Fig. 6(c).

**Figure 7:**
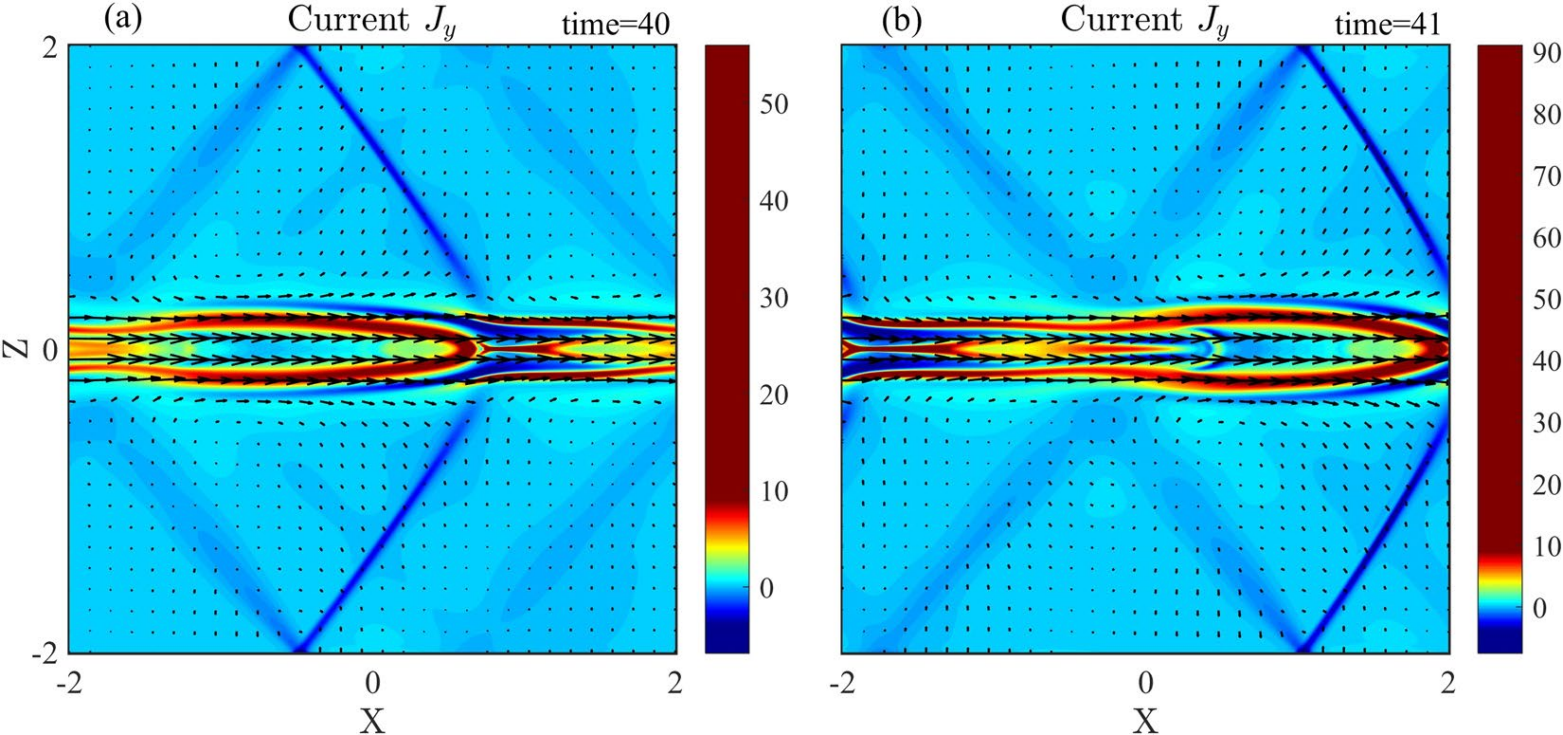
Color contours of the current density (J_y_) with magnetic field lines (black solid lines) and flow vectors (black arrow) at the times: (**a**) t = 40 and (**b**) t = 41.

In order to identify the property of the discontinuities at the nonlinear growth phase, the current density (J_y_) distributions at t = 40 and t = 41 are shown in Fig. 7. The discontinuities and the magnetic island are propagating in the *x* direction. Using the same method as for Fig. 2, we obtain the moving speed (v_d_) of the discontinuity in the inflow region, v_d_ = 1.3v_A_ at t = 40.

**Figure 8:**
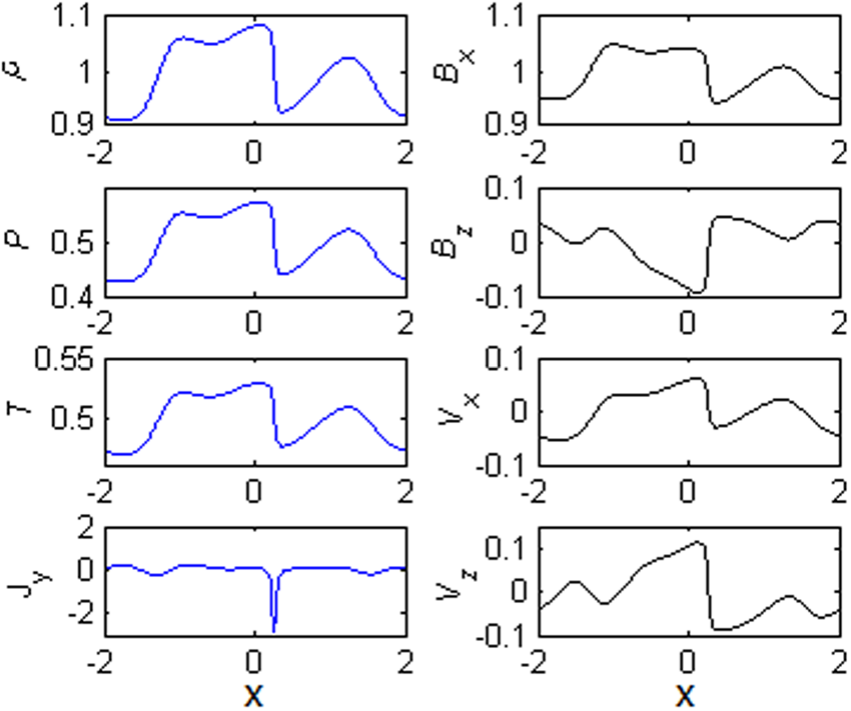
The profiles of the plasma density (ρ), the plasma pressure (P), the plasma temperature (T), the current density (J_y_), the magnetic field (B_x_ and B_z_), and plasma velocity (v_x_ and v_z_) along the x axis at z = 1 for t = 40.

Figure 8 shows the profiles of different physical quantities at z = 1 for t = 40. It is evidently seen that quantities exhibit slow changes about x = −1.4 and sharp variations around x = 0.2, which means that there are multi-pair discontinuities in the inflow region. But, we only analyze the properties of the strong discontinuity layer around x = 0.2. By the transformation of the variables in the upstream and downstream regions into the De Hoffmann-Teller frame, the measured values of the variables in the upstream and downstream regions relative to the discontinuity, as well as that calculated from the Rankine-Hugoniot relations, are given in Table 2. The angle of the discontinuity with respect to the x axis is about 122^0^ at t = 40 in Fig. 7. The characteristic speeds of the slow, intermediate, and fast modes are, respectively, v_sl1_ = 0.6634, v_I1_ = 0.8537, and v_F1_ = 1.1459 in the upstream region, and v_sl2_ = 0.6088, v_I2_ = 0.7974, and v_F2_ = 1.2281 in the downstream region. Comparing with the normal components of the plasma flow velocity relative to the discontinuity in the upstream and downstream in Table 2, we find v_n1_ > v_F1_, v_I2<_v_n2_ < v_F2_. The other variables satisfy the following conditions, $[\rho ] > 0,[{\rm{P}}] > 0,[{v}_{n}] < 0,[|{B}_{t}|] > 0$. It can be concluded that the strong discontinuity in the inflow region as shown in Fig. 7 is corresponding to fast shock.

**Table 2:** The variables from the Rankine-Hugoniot relations for the shock at z = 1 for t = 40 in Fig. 8.

	*ρ*	*P*	*B*_*n*_	*B*_*t*_	*v*_*n*_	*v*_*t*_
Upstream	0.9257	0.4404	0.8214	−0.4629	1.1733	0.6312
Downstream	1.0800	0.5697	0.8287	−0.6269	0.9921	0.7503
R-H matching	1.0238	0.5149	0.8214	−0.5724	1.0609	0.7136
Error	5.2%	9.62%	0.74%	8.69%	6.93%	4.89%

**Figure 9:**
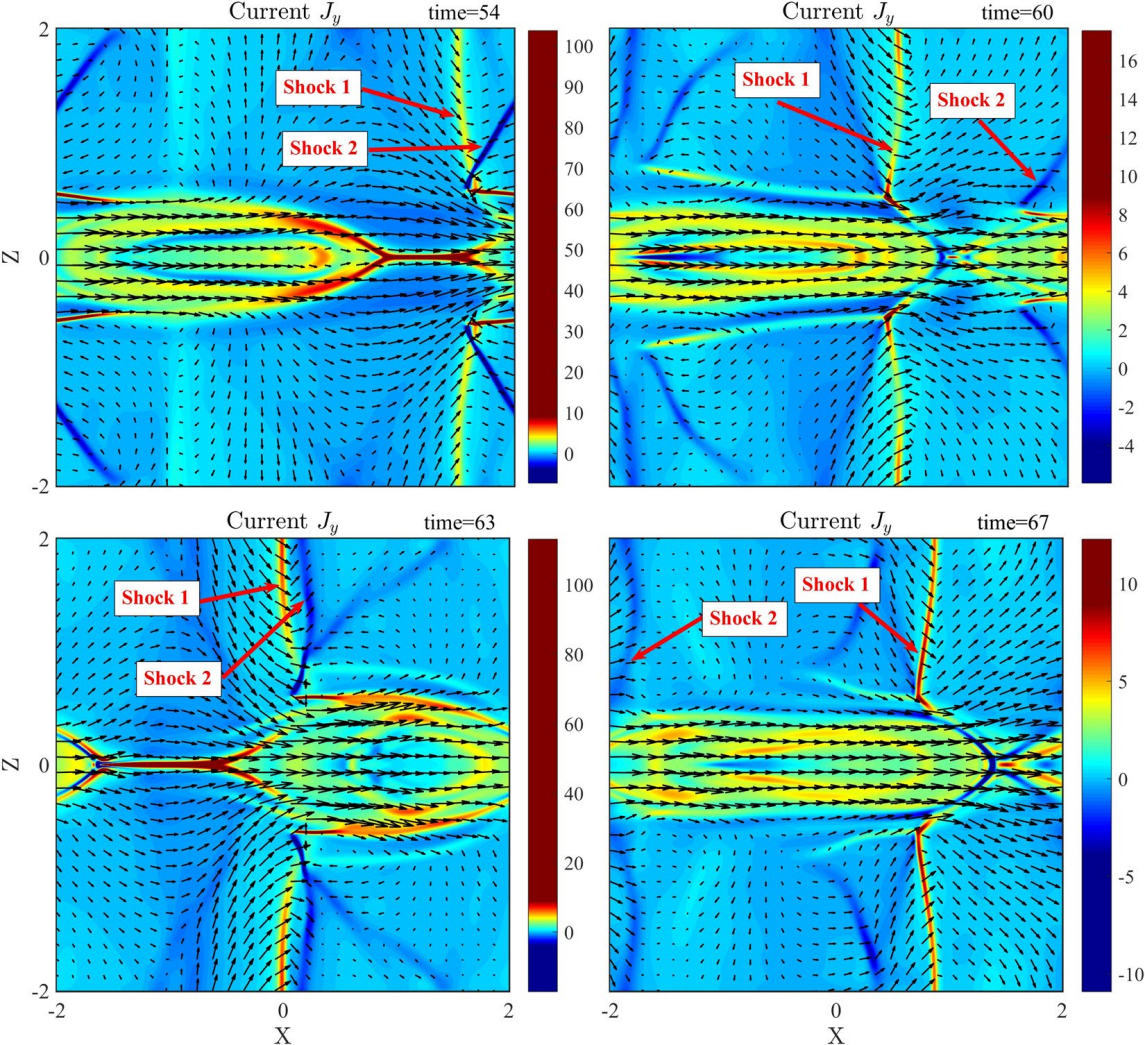
Color contours of the current density (J_y_) with magnetic field lines (black solid lines) and flow vectors (black arrow) at t = 54, 60, 63, and 67, corresponding to d, e, f, and g labeled in Fig. 5, respectively.

Figure 9 shows the current density (J_y_) distributions at t = 54, 60, 63, and 67. It can be seen that there exist multi-pairs of shocks in the inflow region. We label two dominant pairs of shocks as Shock 1 and 2 that are crucial on dynamic evolution of magnetic reconnection. It is clear that Shock 1 is propagating in the *x* direction while Shock 2 is nearly stationary relative to the X point. As we know, shocks can partially block plasma flow. Therefore, shocks how to affect magnetic reconnection depend on their locations. With propagation of Shock 1, two shocks at t = 54 and 63 become very close to each other and both are located in one side of the X point in the inflow region. Thus, the shocks block one side of the plasma inflow entering into the reconnection region and only affect weakly on magnetic reconnection. Thus, magnetic reconnection increases quickly and reaches its peak as shown in Fig. 5

. But at t = 60 and 67, the shocks become separately and are located in two sides of and closely to the X point, which blocks both sides of plasma flow entering into the reconnection region, or magnetic reconnection is suppressed. Thus, the location change of Shock 1 with respect to the X point leads to the quasi-period oscillation of reconnection rate.

**Figure 10:**
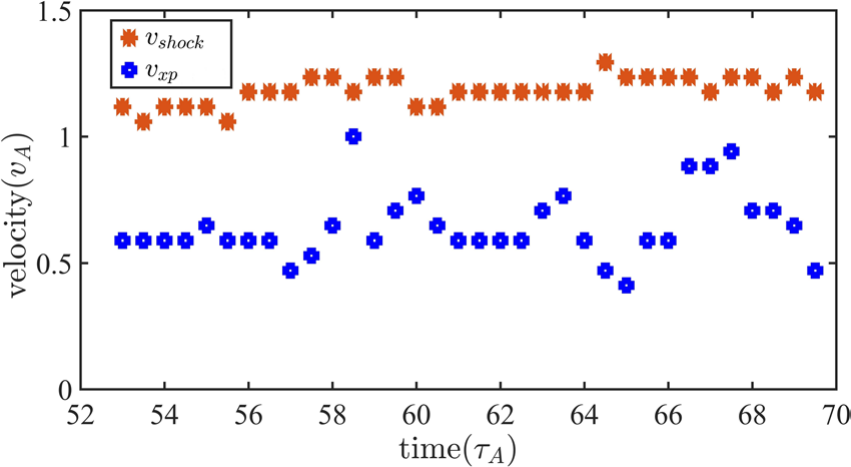
Velocities of Shock 1 and the X point.

Since the oscillating behavior of the magnetic reconnection is associated with the relative locations of Shock 1 and the X point, the oscillating period can be estimated from the relative velocities of Shock 1 and the X point. As shown in Fig. 10, the velocities of Shock 1 and the X point are around 1.2 and 0.7 in average, respectively. The oscillation period is estimated about $2{L}_{x}/({v}_{shock}-{v}_{xp}) \sim 8$ that agrees with the period observed in the simulation. When two pairs of the shocks are located in two sides of the X point, plasma flow is convergent into the current sheet with the *x* direction. Therefore, the velocity of the X point suddenly increases close to the velocity of Shock 1 around t = 58.5 and 67.

## Summary

Compressible resistive MHD model is used to examine formation of shocks in magnetic reconnection with a streaming flow inside the current sheet. We mainly focus on the evolution of shocks in the inflow region and dynamics of magnetic reconnection with sub (super)-Alfvénic streaming flow. It is found that magnetic reconnection develops much faster with the super-Alfvénic streaming flow than with the sub-Alfvénic streaming flow. The time to reach the maximum reconnection rate takes about 100 *τ*_*A*_ for the sub-Alfvénic streaming flow while it only takes about 40 *τ*_*A*_ for the super-Alfvénic streaming flow. The maximum reconnection rate is almost four times larger with the super-Alfvénic streaming flow than with the sub-Alfvénic streaming flow, which suggests that the super-Alfvenic streaming flow can largely enhance magnetic reconnection due to the sausage instability as suggested Lee *et al*.^24^ We also find that the slow shocks are formed by sub-Alfvénic streaming flow and fast shocks by super-Alfvénic streaming flow in the inflow region or outside the reconnection separatrices. The reconnection rate in the decay phase shows a quasi-period oscillation behavior for super-Alfvénic streaming flow, which is resulted from the location change of Shock 1 with respect to the X point due to different propagation speeds of Shock 1 and the X point.
